# Study on the correlation between hyperuricemia and components of metabolic syndrome in T2DM in China: a single-center, observational study

**DOI:** 10.3389/fendo.2026.1697721

**Published:** 2026-03-11

**Authors:** Xiao-fen Lian, Jia-yu Huang, Dong-hui Lu

**Affiliations:** 1 Department of Endocrinology, Peking University Shenzhen Hospital, Shenzhen, Guangdong, China; 2 Shantou University Medical College, Shantou, Guangdong, China

**Keywords:** components, hyperuricemia, metabolic syndrome, serum uric acid, T2DM

## Abstract

**Background:**

Hyperuricemia (HUA) and metabolic syndrome (MetS) are increasingly prevalent and closely related. This single-center, retrospective study aims to explore the correlation between HUA and individual MetS components in Chinese patients with type 2 diabetes mellitus (T2DM), and further explores its potential influence on bone metabolism indicators.

**Method:**

This retrospective study included patients age 18–80 attending Peking University Shenzhen Hospital from March 1, 2023 to May 31, 2024. 426 HUA patients and 1437 normouricemia (NUA) patients were collected and completed clinical and biochemical measurements. Spearman correlation, logistic regression, multiple linear regression, and subgroup analysis were used to verify the relationship between HUA and MetS components (including obesity, hypertension, hyperglycemia, and dyslipidemia). A nomogram and ROC curve were established to identify predictors of HUA risk.

**Result:**

The prevalence of HUA was 22.86%. Spearman analysis showed that BMI (r = 0.322) and TG (r = 0.304) had moderate positive correlations with SUA; sex [female%] exhibited a moderate inverse correlation (r = –0.444). HDL-C (r = –0.218) and eGFR (r = –0.283) showed weak inverse correlations. N-MID (r = –0.049) and P1NP (r = –0.063) showed negligible correlations with SUA, despite statistical significance. Binary logistic regression identified female sex, HbA1c, and eGFR as factors associated with lower HUA risk, while BMI, DBP, TG, and 25(OH)D were associated with increased risk. ROC analysis demonstrated moderate predictive value for BMI (AUC = 0.647), DBP (AUC = 0.602), and 25(OH)D (AUC = 0.578) (all P < 0.001). TG showed an AUC of 0.650 but was not statistically significant (P = 0.502). eGFR (AUC = 0.364) and HbA1c (AUC = 0.489) performed worse than random chance. Multiple linear regression revealed weak positive associations of SUA with BMI (β = 0.211) and TG (β = 0.151), weak inverse associations with HbA1c (β = –0.153) and HDL-C (β = –0.160), and a negligible association with 25(OH)D (β = 0.055). Subgroup analysis indicated higher HUA risk in males and those with metabolic abnormalities.

**Conclusion:**

This exploratory study identifies moderate predictive value of BMI, DBP, and 25(OH)D for HUA in Chinese T2DM patients, and a substantially lower risk in females. TG was not an independent predictor. Associations with bone markers and 25(OH)D were negligible. These findings highlight modest interconnections between HUA and metabolic dysregulation, particularly obesity.

## 1 Introduction

In recent years, as lifestyle and dietary patterns have changed, the global prevalence of hyperuricemia (HUA) has significantly increased, showing a younger change and posing a substantial public health challenge (1, 2). Epidemiological studies indicate that in certain developed countries, the prevalence exceeds 20% (3),while some regions in China report similar rates (4). HUA is a metabolic disease caused by abnormal purine metabolism, which leads to excessive production or reduced excretion of SUA in the body, resulting in its elevated serum levels. Recent research proves that HUA not only triggers of gout and gouty arthritis, causing joint deformity and dysfunction (5), but is also strongly associated with systemic conditions such as kidney disease (6), cardiovascular disorders (7), cancer (8), stroke (9).

MetS refers to a cluster of metabolic abnormalities, including obesity, hypertension, hyperglycemia, and dyslipidemia (10). Its primary cause lies in the disruption of metabolic signaling pathways due to insulin resistance (11), which leads to glucose regulation imbalance, lipid metabolism disturbances, and vascular endothelial dysfunction (12). The presence of MetS substantially increases the risk of type 2 diabetes mellitus (T2DM) (13), cardiovascular disease (14), and cerebrovascular disease (15), thereby compromising human health and quality of life.

Recently, the association between HUA and MetS has attracted extensive attention. Numerous studies reveal that the incidence of MetS in individuals with HUA rises markedly with increasing SUA levels (16–19),Even within the normal range of SUA, there exists a dose-dependent positive correlation with MetS risk (19). Notably, this relationship is bidirectional, as patients with MetS exhibit an elevated risk of HUA. Further analyses demonstrate that elevated SUA levels are independently associated with MetS components, such as obesity, hypertension, and hyperlipidemia (20–22), making it a robust and independent predictor of MetS. This association remains statistically significant after adjusting for confounding variables (23). However, a study involving 1,381 healthy Chinese women using Mendelian randomization did not identify elevated SUA as a causal factor for MetS or its components (24). Additionally, the potential influence of HUA on bone metabolism indicators may indirectly contribute to the pathological network of MetS, although this effect hasn’t been systematically integrated and studied. SUA and bone metabolism have a complex, dual relationship. Under normal physiological conditions, SUA may exert bone-protective effects. In contrast, in HUA or gout, elevated SUA may exacerbate bone resorption, inhibit bone formation, and increase fracture risk by promoting inflammation and oxidative stress (25).

While existing studies generally confirm the strong link between HUA and MetS components, comprehensive data specifically in Chinese patients with T2DM, particularly analyses incorporating bone metabolism markers, remain limited. This single-center, retrospective study therefore aims to conduct an exploratory analysis of the associations between HUA and individual components of MetS in this population, and to descriptively examine its relationship with bone metabolism markers. Given its exploratory nature, the study is positioned to generate hypotheses rather than to confirm definitive causal pathways. We employ multiple statistical models to identify metabolic factors associated with HUA and evaluate their predictive performance, with explicit interpretation of effect sizes according to established thresholds. The findings are expected to offer preliminary insights and inform future hypothesis-driven research on risk assessment and management of patients with T2DM and concomitant metabolic abnormalities.

## 2 Materials and methods

### 2.1 Study population and design

This was a retrospective study. The study comprised participants aged 18–80 years admitted to Peking University Shenzhen Hospital between March 1, 2023 to May 31, 2024. Clinical assessment, and biochemical measurements were obtained from 426 HUA patients and 1,437 NUA patients (1125 men and 738 women). HUA is defined as a fasting SUA level of more than 420 μmol/L after normal daily diet on two different days. (26). Patients were excluded if they had malignant tumors, acute infectious diseases, kidney diseases, or were taking medications (such as diuretics or allopurinol) known to influence SUA levels. Ethical approval was granted with exemption due to the retrospective nature of the study. All participants provided informed consent upon admission for the use of their health information in medical research.

### 2.2 Data collection

The basic information, clinical assessment, and biochemical measurements of all subjects were collected through the hospital information system (HIS). The data included age, gender, weight, height, body mass index (BMI), systolic blood pressure (SBP), diastolic blood pressure (DBP), hemoglobin A1c (HbA1c), fasting plasma glucose (FPG), total cholesterol (TC), triglycerides (TG), low-density lipoprotein cholesterol (LDL-C), high-density lipoprotein cholesterol (HDL-C), alanine aminotransferase (ALT), SUA (SUA), creatinine (Cr), estimated glomerular filtration rate (eGFR), thyroid stimulating hormone (TSH), 25-hydroxyvitamin D (25(OH)D),alkaline phosphatase (ALP), N-terminal osteocalcin (N-MID), type 1 collagen N-terminal peptide (P1NP).

### 2.3 Clinical assessment

Well-trained nurses measured each participant’s weight, height, and blood pressure upon admission. Participants’ weight and height were measured to the nearest 0.1 kg and 0.1 cm, respectively, while wearing light clothing and no shoes. The body mass index (BMI) was computed by dividing weight in kilograms (kg) by height in meters squared (m). SBP and DBP in the right upper arm were measured using a validated digital automatic analyzer (Omron, Japan) in a seated position after at least 5 minutes of rest, with an additional recording after a 2-minute pause. SBP and DBP were both measured twice, and the average of the two measurements was used for analysis. If the two readings differed by >5 mmHg, a third measurement was performed, and the average of all three readings was applied.

### 2.4 Biochemical measurements

All enrolled participants had their blood samples collected in the morning after fasting for at least 8 hours during hospitalization. The laboratory’s key outcome parameters were blood lipids (TC, TG, LDL-C, HDL-C) and glucose metabolism indicators (HbA1c, FPG). Plasma glucose was tested using the standard hexokinase technique. HbA1c was determined using capillary electrophoresis. TG, TC, LDL-C and HDL-C were determined using enzymatic assays (GK-GPO-POD colorimetric method), cholesterol oxidase methods, and homogeneous methods, respectively. All of the parameters listed above were determined using an automatic biochemical analyzer (AU5800 Series Chemistry Analyzers, Beckman Coulter) in the clinical laboratory of Peking University Shenzhen Hospital.

### 2.5 Definition of outcomes

The components of MetS examined in this study included BMI, SBP, DBP, glucose metabolism-related indicators (including FPG and HbA1c), and lipid profiles (including TC, TG, LDL-C, and HDL-C).

The diagnosis of MetS was made using the criteria recommended by the Chinese Medical Association Diabetes Division (CDS) in 2004 (27): (1) Overweight and/or Obesity: BMI ≥25.0kg/m^2^; (2) Hyperglycemia: FPG ≥6.1 mmol/L (110mg/dL), 2hPG ≥7.8 mmol/L (140mg/dL), and/or diagnosis of and treatment for diabetes; (3) Hypertension: SBP/DBP ≥140/90 mmHg and/or confirmation and treatment of hypertension; (4) Dyslipidemia: TG ≥1.7 mmol/L (150mg/dL) and/or HDL-C <0.9 mmol/L (35mg/dL) (male) or <1.0 mmol/L(39mg/dL) (female). MetS was diagnosed when 3 or more of the above 4 components met the criteria.

### 2.6 Statistical analysis

Participants were classified by SUA levels, and baseline characteristics for all continuous variables meeting normality assumptions were presented as means ± standard deviation (SD). To compare the differences between HUA and NUA groups, Student’s t-test was performed for continuous variables. Spearman correlation analysis was used to analyze whether SUA concentrations linked to sex, age, BMI, SBP, DBP, FPG, HbA1c, TC, TG, LDL-C, HDL-C, ALT, eGFR, h-TSH, 25(OH)D, ALP, N-MID, P1NP. Furthermore, binary logistic regression analysis was used to determine the independent predictors of HUA risk. The independent variables in binary logistic regression were used to establish a risk prediction model for HUA. Based on Akaike information criterion (AIC), a stepwise logistic regression equation was constructed, and a visual nomogram was constructed. ROC curve was used to analyze the diagnostic efficacy of each parameter in predicting HUA. To determine whether SUA levels independently affected the above indicators, three models were used for multiple linear regression analysis: Model 1 (unadjusted), Model 2 (adjusted for ALT and eGFR), Model 3 (adjusted for age, sex, ALT, and eGFR), and the predicted and observed values of SUA levels were plotted based on the linear regression model. Finally, to assess whether the association between SUA and metabolic parameters differed across subgroups, subgroup analyses were performed. All statistical analyses were performed using SPSS 26.0 and R 4.1.3 software.

All statistical tests were two-sided, with P < 0.05 considered significant. Given the exploratory nature and multiple comparisons, p-values were interpreted as hypothesis-generating, and no multiplicity adjustment was applied. Effect sizes were evaluated using established thresholds: For |r| or |β|, <0.10 = negligible, 0.10–0.30 = weak, 0.30–0.50 = moderate, ≥0.50 = strong (adapted from Cohen, 1988). The sample size (n = 1,863) provided adequate power to detect weak-to-moderate associations in correlation and regression analyses.

## 3 Result

### 3.1 Characteristics of the study population

A total of 1,863 participants were enrolled in the study, with 426 in the HUA group and 1,437 in the NUA group. The characteristics of the population in both groups are presented in **Table 1**. Overall, the prevalence of HUA was 22.86%. Significant differences were observed between both groups across various metabolic and physiological parameters. The HUA group had a significantly lower proportion of female (7.59% vs. 47.45%, P<0.0001) and slightly lower age (53.42 ± 10.38 vs. 54.56 ± 10.02, P = 0.0414) compared to the NUA group. The metabolic markers (BMI, SBP, DBP, TG, TC, LDL-C, IDL-C, ALT, 25(OH)D) were considerably higher in the HUA group (P<0.05). The HUA group showed significantly reduced levels of HbA1c, HDL-C, eGFR, and bone formation markers (N-MID, P1NP) compared to the control group (P<0.05). There were no significant differences in FPG, h-TSH, or ALP between the two groups (P > 0.05).

**Table 1:** Baseline characteristics of the study population stratified by SUA status.

Characteristics	NUA (N = 1,437)	HUA (N = 426)	P value
Sex [Female, n (%)]	682(47.45%)	56(7.59%)	**<0.0001**
Age (years)	54.56 ± 10.02	53.42 ± 10.38	**0.0414**
BMI (kg/m²)	24.1 ± 2.79	25.48 ± 2.79	**<0.0001**
SBP (mmHg)	121.74 ± 17.35	125.3 ± 17.01	**0.0002**
DBP (mmHg)	73.06 ± 10.57	77.15 ± 10.78	**<0.0001**
FPG (mmol/L)	6.04 ± 2.18	5.83 ± 1.89	0.0778
HbA1c (%)	6.55 ± 1.84	6.27 ± 1.48	**0.0048**
TG (mmol/L)	1.54 ± 0.99	2.02 ± 1.21	**<0.0001**
TG/FPG	0.27 ± 0.17	0.36 ± 0.21	**<0.0001**
TC (mmol/L)	5.16 ± 1.17	5.4 ± 1.14	**0.0001**
LDL-C (mmol/L)	3.37 ± 0.9	3.59 ± 0.83	**<0.0001**
HDL-C (mmol/L)	1.29 ± 0.32	1.19 ± 0.28	**<0.0001**
IDL-C (mmol/L)	0.5 ± 0.31	0.62 ± 0.42	**<0.0001**
ALT(U/L)	21.32 ± 11.26	26.2 ± 13.24	**<0.0001**
eGFR(mL/min/1.73^2)	105.23 ± 23.52	95.39 ± 19.11	**<0.0001**
h-TSH (mIU/L)	2.16 ± 1.31	2.13 ± 1.28	0.6688
25(OH)D (nmol/L)	60.25 ± 20.28	65.83 ± 21.40	**<0.0001**
ALP (U/L)	82.27 ± 22.54	81.25 ± 22.06	0.4342
N-MID (ng/ml)	16.82 ± 6.52	15.86 ± 5.61	**0.0057**
P1NP (ng/ml)	47.77 ± 19.77	44.13 ± 16.45	**0.0006**

*Data are presented as the Mean (SD) or Median (IQR) for continuous variables and percentage (%) for categorical variables.

*The Student’s t-test was used for continuous variables, and the Chi-square test was used for categorical variables.

*The bold values mean their value less than 0.05, which is statistically significant (i.e., P<0.05).

*SUA, serum uric acid; HUA, hyperuricemia; NUA, normouricemia; BMI, body mass index; SBP, systolic blood pressure; DBP, diastolic blood pressure; FPG, fasting plasma glucose; HbA1c, hemoglobin A1c; TG, triglycerides; TC, total cholesterol; LDL-C, low-density lipoprotein cholesterol; HDL-C, high-density lipoprotein cholesterol; IDL-C, intermediate-density lipoprotein cholesterol; ALT, alanine aminotransferase; eGFR, estimated glomerular filtration rate; h-TSH, human thyroid-stimulating hormone; 25(OH)D, 25-hydroxyvitamin D; ALP, alkaline phosphatase; N-MID, N-terminal mid-fragment of osteocalcin; P1NP, N-terminal propeptide of type I procollagen.

### 3.2 Correlation analysis of SUA levels with clinical and biochemical parameters

Spearman correlation analysis revealed significant associations between SUA concentrations and various parameters (**Table 2**). Moderate positive correlations with SUA were observed for BMI (r = 0.322, P < 0.001) and TG (r = 0.304, P < 0.001). Weak positive correlations were noted for DBP (r = 0.167, P < 0.001), TC (r = 0.111, P < 0.001), LDL-C (r = 0.146, P < 0.001), ALT (r = 0.282, P < 0.001), and 25(OH)D (r = 0.143, P < 0.001). Although SBP (r = 0.094, P < 0.001) reached statistical significance, its effect size bordered on trivial, indicating limited clinical relevance. Regarding inverse associations, a clear inverse correlation of moderate magnitude was found for sex [Female%] (r = –0.444, P < 0.001). HDL-C (r = –0.218, P < 0.001) and eGFR (r = –0.283, P < 0.001) demonstrated modest negative correlations, falling within the small-to-moderate range. Of note, the bone metabolism markers N-MID (r = –0.049, P = 0.035) and P1NP (r = –0.063, P = 0.007) achieved nominal significance, yet their effect sizes were trivial, suggesting negligible linear associations with minimal clinical meaningfulness. In addition, no significant correlations were observed between UA and age, FPG, HbA1c, or ALP (P>0.05).

**Table 2:** Spearman correlation analysis between SUA levels and clinical parameters in all participants.

*Characteristics*	SUA(μmol/L)	
	*r*	*P value*
Sex [Female, n (%)]	-0.444	**<0.001**
Age (years)	-0.026	0.260
BMI (kg/m²)	0.322	**<0.001**
SBP (mmHg)	0.094	**<0.001**
DBP (mmHg)	0.167	**<0.001**
FPG (mmol/L)	0.023	0.319
HbA1c (%)	0.014	0.551
TG (mmol/L)	0.304	**<0.001**
TC (mmol/L)	0.111	**<0.001**
LDL-C (mmol/L)	0.146	**<0.001**
HDL-C (mmol/L)	-0.218	**<0.001**
ALT(U/L)	0.282	**<0.001**
eGFR(mL/min/1.73^2)	-0.283	**<0.001**
h-TSH (mIU/L)	-0.008	0.734
25(OH)D (nmol/L)	0.143	**<0.001**
ALP (U/L)	0.020	0.412
N-MID (ng/ml)	-0.049	**0.035**
P1NP (ng/ml)	-0.063	**0.007**

*P value determined by spearman correlation analysis with respect to the SUA level.

*The bold values mean their value less than 0.05, which is statistically significant (i.e., P<0.05).

*SUA, serum uric acid; body mass index; SBP, systolic blood pressure; DBP, diastolic blood pressure; FPG, fasting plasma glucose; HbA1c, hemoglobin A1c; TG, triglycerides; TC, total cholesterol; LDL-C, low-density lipoprotein cholesterol; HDL-C, high-density lipoprotein cholesterol; IDL-C, intermediate-density lipoprotein cholesterol; ALT, alanine aminotransferase; eGFR, estimated glomerular filtration rate; h-TSH, human thyroid-stimulating hormone; 25(OH)D, 25-hydroxyvitamin D; ALP, alkaline phosphatase; N-MID, N-terminal mid-fragment of osteocalcin; P1NP, N-terminal propeptide of type I procollagen.

### 3.3 Binary logistic regression analysis of SUA levels and clinical and biochemical parameters

**Figure 1:**
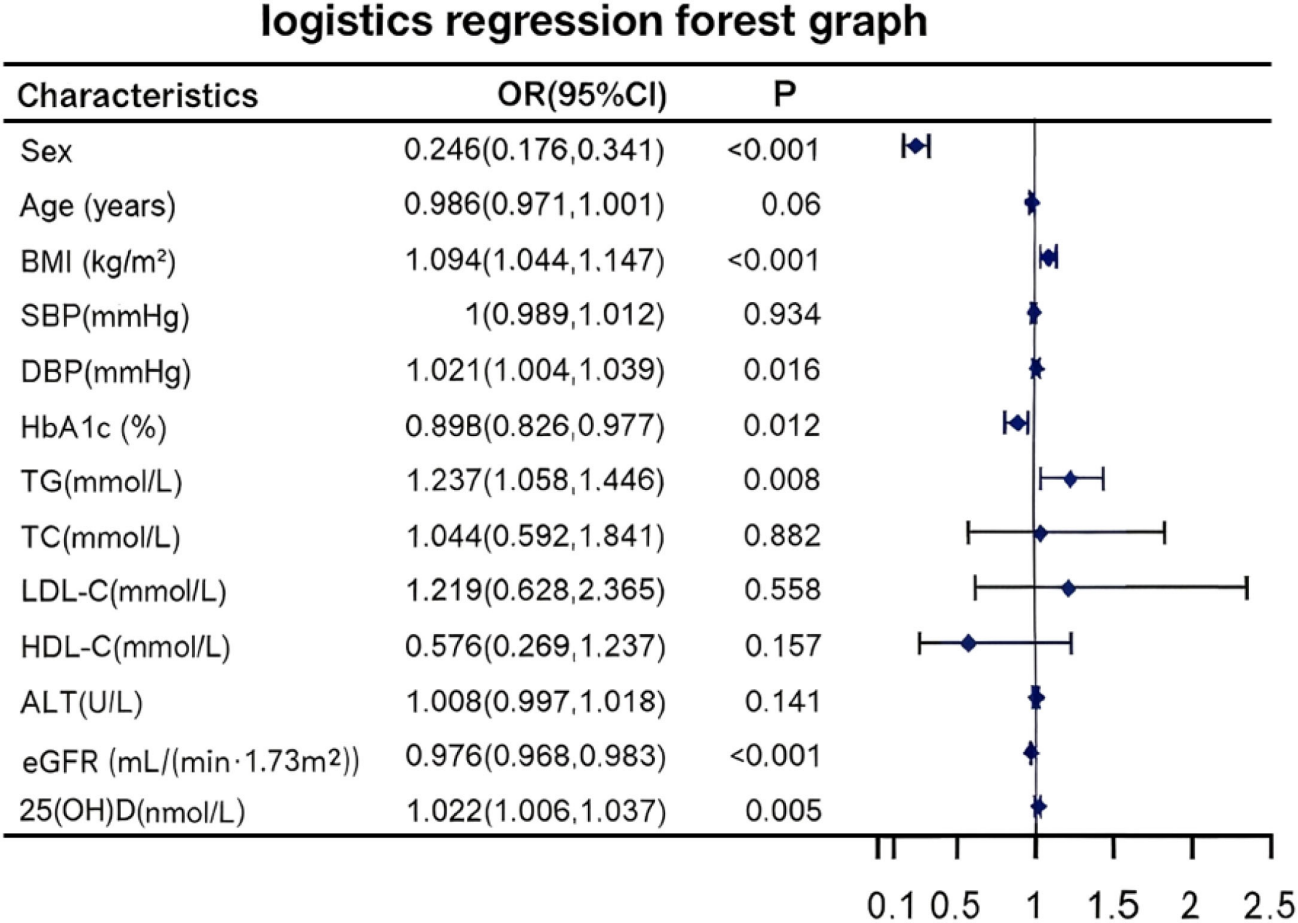
Forest plot of independent risk factors for HUA identified by binary logistic regression. *Odds ratios (ORs) with 95% confidence intervals (CIs) from binary logistic regression analysis show the association between clinical parameters and HUA risk. *An OR > 1 indicates increased risk, while an OR < 1 indicates decreased risk. The vertical dashed line represents the null effect (OR = 1). *P value less than 0.05, which is statistically significant (i.e., P<0.05). *HUA, hyperuricemia; OR, odds ratio; CI, confidence interval; BMI, body mass index; SBP, systolic blood pressure; DBP, diastolic blood pressure; HbA1c, hemoglobin A1c; TG, triglycerides; TC, total cholesterol; LDL-C, low-density lipoprotein cholesterol; HDL-C, high-density lipoprotein cholesterol; ALT, alanine aminotransferase; eGFR, estimated glomerular filtration rate; 25(OH)D, 25-hydroxyvitamin D.

Binary logistic regression analysis identified several independent predictors of HUA risk (**Figure 1**). Compared to males, females had a substantially lower risk of HUA (adjusted OR = 0.245, 95% CI: 0.176–0.341). Elevated BMI (OR = 1.094, 95% CI: 1.044–1.147, P < 0.001) and higher TG (OR = 1.237, 95% CI: 1.058–1.446, P = 0.008) were associated with an increased risk of HUA. Higher DBP (OR = 1.021, 95% CI: 1.004–1.039, P = 0.016) and increased 25(OH)D levels (OR = 1.022, 95% CI: 1.006–1.037, P = 0.005) also showed positive associations with HUA, although the odds ratios were close to 1.0, indicating a minimal increment in risk per unit increase. In contrast, higher HbA1c (OR = 0.898, 95%CI: 0.826–0.977, P = 0.012) and eGFR (OR = 0.976, 95%CI: 0.968–0.983, p<0.001) showed inverse associations with HUA.

### 3.4 Prediction model for HUA

**Figure 2:**
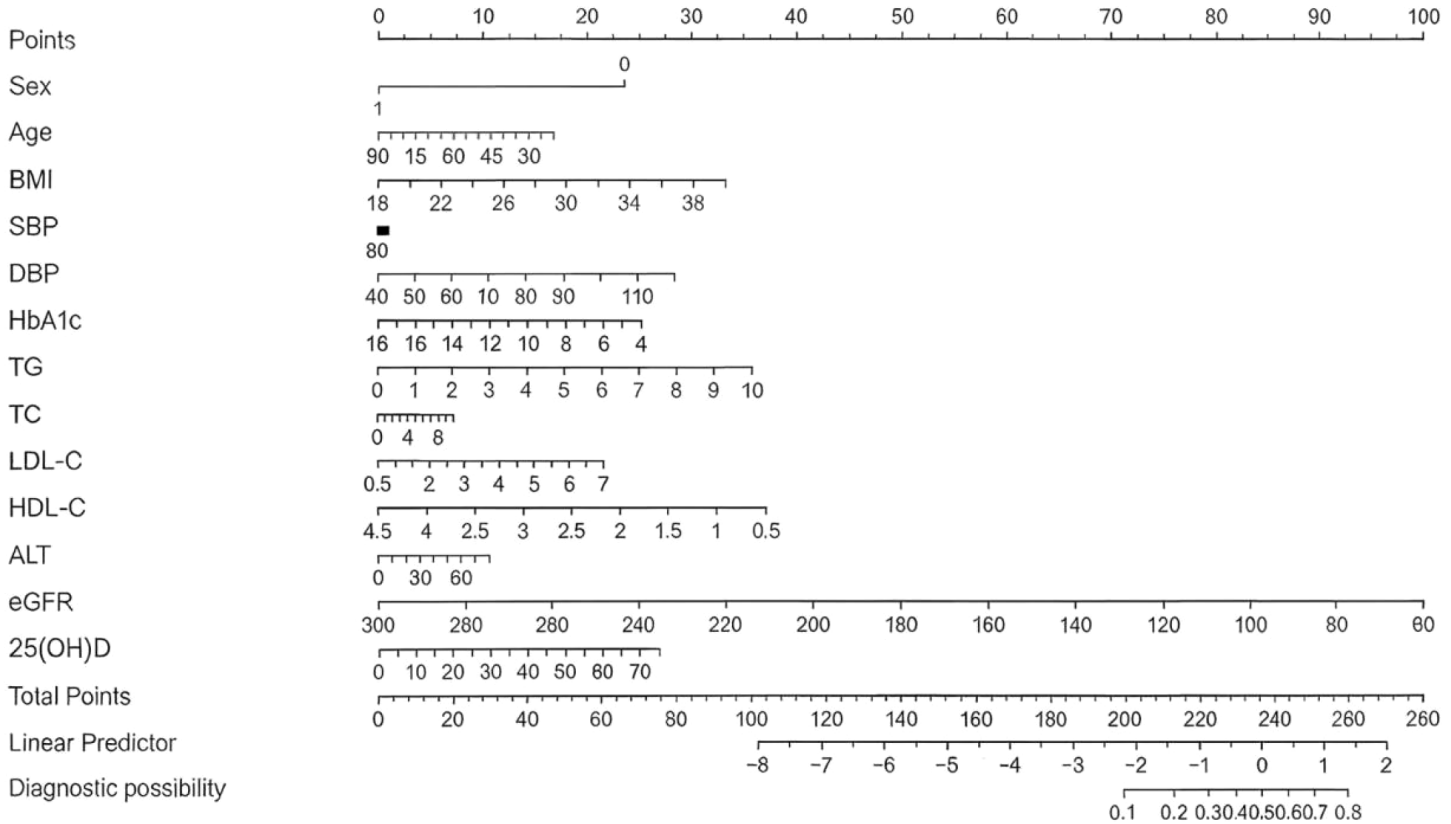
Nomogram for predicting the risk of HUA in Chinese patients with T2DM. * The nomogram was developed based on a binary logistic regression model using stepwise selection, incorporating the following predictors: sex age, BMI, SBP, DBP, FPG, HbA1c, TC, TG, LDL-C, HDL-C, ALT, eGFR and 25(OH)D. *For each predictor, draw a line upward to the ‘Points’ scale to determine its score. Sum all individual scores to obtain the “Total Points”. Finally, project the Total Points downward onto the bottom “Diagnostic possibility” axis to read the predicted probability of HUA. * HUA, hyperuricemia; T2DM, type 2 diabetes mellitus; BMI, body mass index; SBP, systolic blood pressure; DBP, diastolic blood pressure; FPG, fasting plasma glucose; HbA1c, hemoglobin A1c; TC, total cholesterol; TG, triglycerides; LDL-C, low-density lipoprotein cholesterol; HDL-C, high-density lipoprotein cholesterol; ALT, alanine aminotransferase; eGFR, estimated glomerular filtration rate; 25(OH)D, 25-hydroxyvitamin D.

A risk prediction model for HUA was established using the independent variables identified from the binary logistic regression analysis. A stepwise logistic regression equation was constructed based on AIC, and a visual nomogram was created (**Figure 2**). The nomogram included gender, age, BMI, SBP, DBP, FPG, HbA1c, TC, TG, LDL-C, HDL-C, ALT, eGFR and Vit-D. Each predictive factor is assigned a corresponding score, and the cumulative sum of the corresponding scores for all factors was the total score. The total score corresponded to the predicted likelihood, which was the probability of HUA.

**Figure 3:**
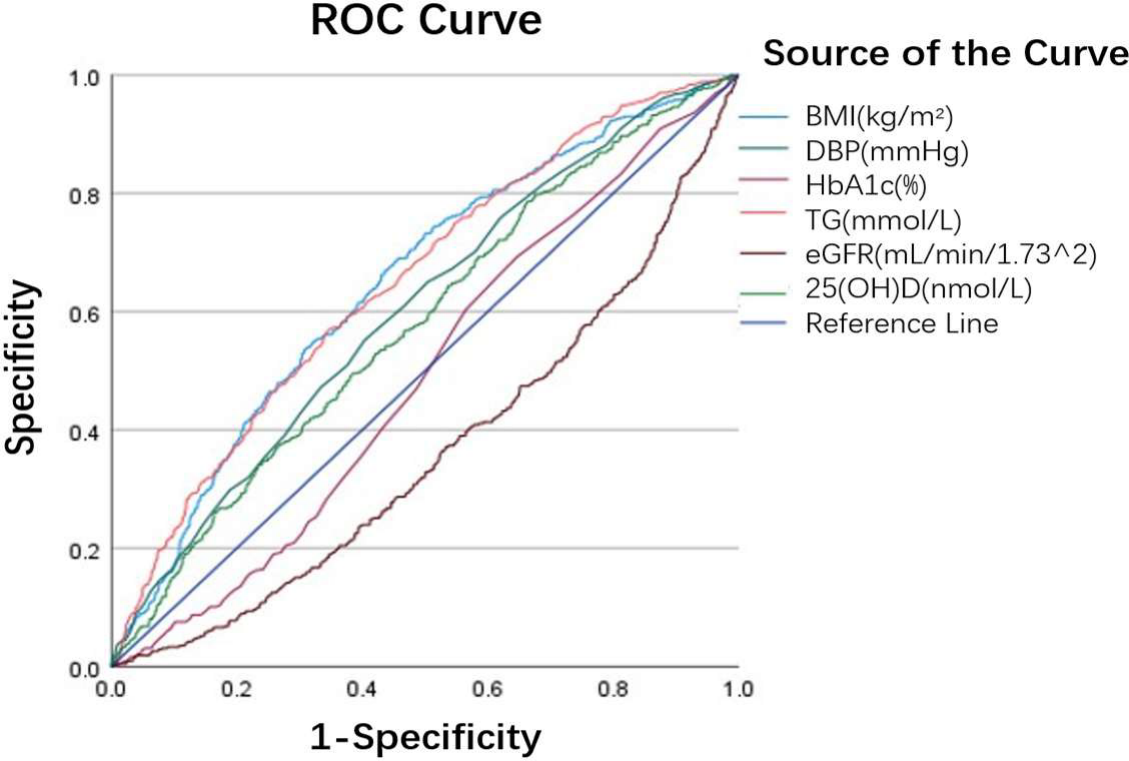
ROC curves for predicting HUA risk in Chinese T2DM patients. *ROC curves illustrating diagnostic performance of BMI, DBP, HbA1c, TG, eGFR, 25(OH)D for HUA. *The area under the curve (AUC) quantifies each parameter’s discriminatory ability: higher AUC values indicate stronger predictive power. The diagonal “Reference Line” represents a random guess (AUC = 0.5). * HUA, hyperuricemia; T2DM, type 2 diabetes mellitus; ROC, receiver operating characteristic; AUC, area under the curve; BMI, body mass index; DBP, diastolic blood pressure; TG, triglycerides; eGFR, estimated glomerular filtration rate; 25(OH)D, 25-hydroxyvitamin D.

The diagnostic performance of related variables in predicting HUA was evaluated using receiver operating characteristic (ROC) curve analysis (**Figure 3**, **Table 3**). BMI (AUC = 0.647, P < 0.001), DBP (AUC = 0.602, P < 0.001), and 25(OH)D (AUC = 0.578, P < 0.001) each demonstrated moderate predictive value for HUA. TG exhibited an AUC of 0.650, but this did not reach statistical significance (P = 0.502); therefore, it was not considered to have independent predictive value. eGFR(AUC = 0.364, P < 0.001)and HbA1c(AUC = 0.482, P < 0.001)both exhibited AUC values below 0.50, indicating discriminative performance worse than random guessing and thus no practical utility as standalone predictors for HUA.

**Table 3:** Data related to variables of ROC curve.

Characteristics	AUC	P	Point of diagnosis	Sensitivity	1-Sensitivity	Specificity
BMI (kg/m2)	0.647	**<0.001**	24.36	0.669	0.436	0.564
DBP (mmHg)	0.602	**<0.001**	75.50	0.552	0.404	0.596
HbA1c (%)	0.489	**<0.001**	5.55	0.692	0.646	0.354
TG (mmol/L)	0.650	0.502	1.56	0.568	0.346	0.654
eGFR (mL/min/1.73^2)	0.364	**<0.001**	60.54	1.000	0.999	0.001
25(OH)D (nmol/L)	0.578	**<0.001**	49.95	0.786	0.663	0.337

*Point of diagnosis = optimal diagnostic threshold for predicting HUA; Sensitivity = proportion of true HUA cases correctly identified; Specificity = proportion of true NUA cases correctly identified; 1-Sensitivity = false negative rate.

*The bold values mean their value less than 0.05, which is statistically significant (i.e., P<0.05).

*ROC, receiver operating characteristic; AUC, area under the curve; BMI, body mass index; DBP, diastolic blood pressure; HbA1c, hemoglobin A1c; TG, triglycerides; eGFR, estimated glomerular filtration rate; 25(OH)D, 25-hydroxyvitamin D.

### 3.5 Multiple linear regression analysis of SUA levels and metabolic parameters

**Figure 4:**
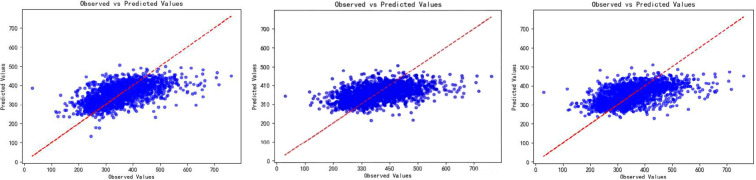
Scatter plots of observed versus predicted SUA levels from three multiple linear regression models. *Model 1: unadjusted; Model 2: adjusted for ALT and eGFR; Model 3: adjusted for age, sex, ALT, and eGFR. *Scatter plots comparing observed SUA levels (x-axis) with predicted SUA levels (y-axis) from three sequential multiple linear regression models. *Each point represents an individual patient. The proximity of points to the diagonal (ideal fit line) indicates the model’s goodness of fit, with closer clustering reflecting stronger agreement between observed and predicted SUA values. * SUA, serum uric acid; ALT, alanine aminotransferase; eGFR, estimated glomerular filtration rate.

Multiple linear regression analyses demonstrated significant associations between SUA and metabolic parameters (**Table 4**). In unadjusted Model 1, SUA showed weak positive associations with BMI (β = 0.156, P < 0.001) and TG (β = 0.141, P < 0.001), while a trivial positive association was observed with 25(OH)D (β = 0.029, P < 0.001). Inverse associations were also noted: HbA1c (β = –0.083, P < 0.001) and HDL-C (β = –0.079, P = 0.014) both exhibited trivial negative effects. After adjusting for ALT and eGFR (Model 2), the positive associations with BMI (β = 0.175, P < 0.001) and TG (β = 0.150, P < 0.001) remained weak, while the trivial association with 25(OH)D (β = 0.036, P < 0.001) persisted. The inverse associations with HbA1c (β = –0.141, P < 0.001) and HDL-C (β = –0.065, P = 0.050) strengthened to weak and trivial-to-weak levels, respectively. Further adjustment for age and sex (Model 3) amplified the weak positive associations with BMI (β = 0.211, P < 0.001) and TG (β = 0.151, P < 0.001). DBP, which was not significantly associated in Models 1–2, emerged as a weak positive correlate in Model 3 (β = 0.147, P = 0.001). The positive association with 25(OH)D remained statistically significant but its effect size remained trivial (β = 0.055, P < 0.001). Inverse associations with HbA1c (β = –0.153, P < 0.001) and HDL-C (β = –0.160, P < 0.001) both fell into the weak range. SBP shifted from non-significant (Models 1–2, β = –0.006 to –0.001, P > 0.05) to a statistically significant but trivial negative association in Model 3 (β = –0.078, P = 0.008). TC and LDL-C showed no significant associations with SUA in any model (all P > 0.05). The scatter plots of the three models showed that the predicted values were close to the observed values, suggesting that the model fitting effect was good (**Figure 4**).

**Table 4:** Multiple linear regression analysis of associations between SUA levels and metabolic parameters.

Outcomes	Model 1		Model 2		Model 3	
	β	P-value	β	P-value	β	P-value
BMI (kg/m^2^)	0.156	**<0.001**	0.175	**<0.001**	0.211	**<0.001**
Blood pressure						
SBP (mmHg)	-0.006	0.840	-0.001	0.963	-0.078	**0.008**
DBP (mmHg)	0.052	0.069	0.049	0.100	0.147	**<0.001**
Glucometabolic status						
HbAlc (%)	-0.083	**<0.001**	-0.141	**<0.001**	-0.153	**<0.001**
Lipid profile						
TG (mmol/L)	0.141	**<0.001**	0.150	**<0.001**	0.151	**<0.001**
TC (mmol/L)	0.021	0.823	-0.031	0.744	-0.020	0.843
LDL-C (mmol/L)	0.059	0.473	0.127	0.134	0.120	0.181
HDL-C (mmol/L)	-0.079	**0.014**	-0.065	0.050	-0.160	**<0.001**
Bone metabolism						
25(OH)D (nmol/L)	0.029	**<0.001**	0.036	**<0.001**	0.055	**<0.001**

*Model 1: unadjusted; Model 2: adjusted for ALT and eGFR; Model 3: adjusted for age, sex, ALT, and eGFR.

*β represents the standardized regression coefficient.

*The bold values mean their value less than 0.05, which is statistically significant (i.e., P<0.05).

*SUA, serum uric acid; BMI, body mass index; SBP, systolic blood pressure; DBP, diastolic blood pressure; HbA1c, hemoglobin A1c; TG, triglycerides; TC, total cholesterol; LDL-C, low-density lipoprotein cholesterol; HDL-C, high-density lipoprotein cholesterol; 25(OH)D, 25-hydroxyvitamin D.

### 3.6 Subgroup analysis of HUA and related parameters

**Figure 5:**
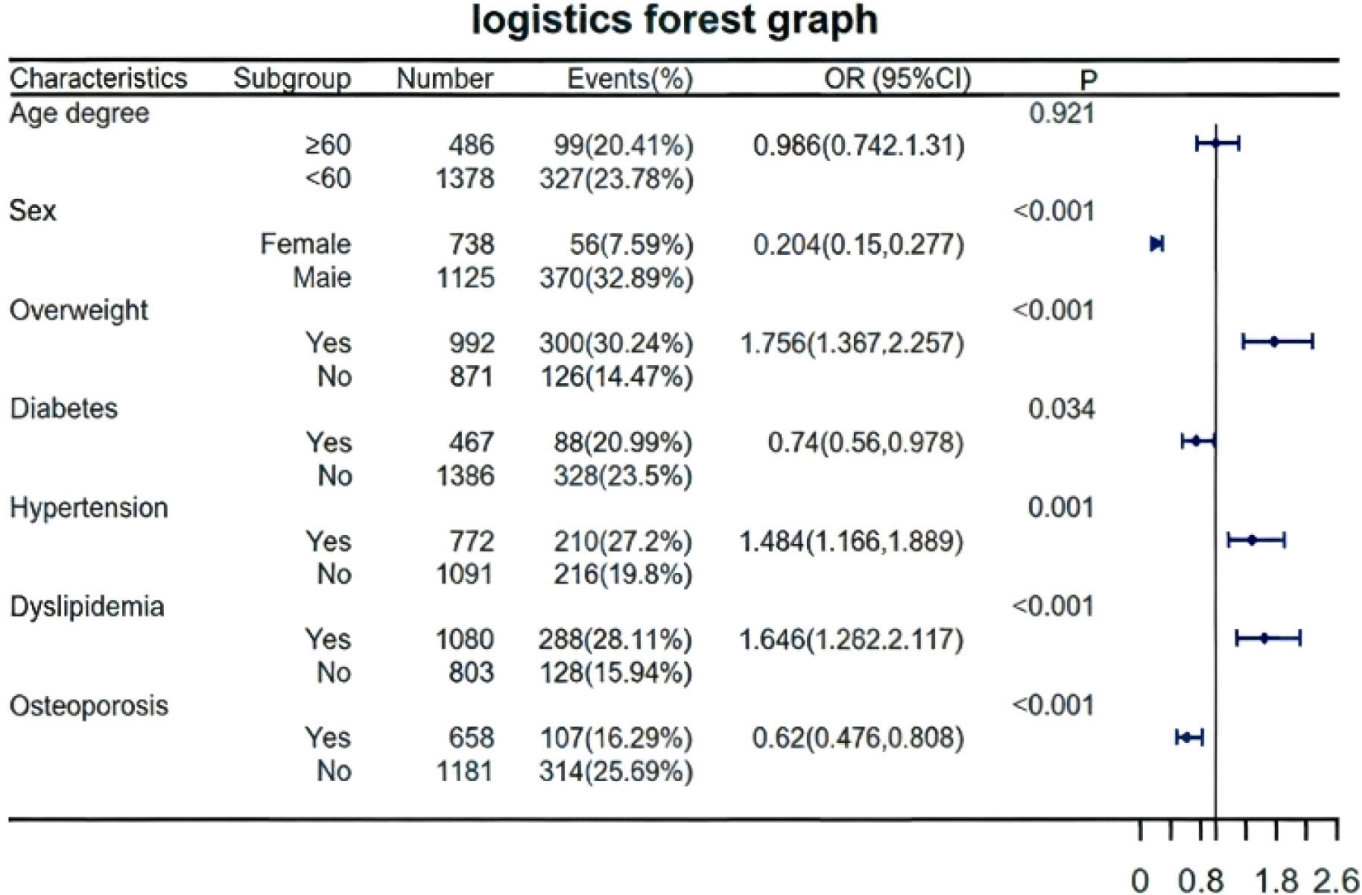
Forest plot of logistic regression and subgroup analysis for HUA risk factors in Chinese T2DM patients. * Forest plot displaying odds ratios (ORs) with 95% confidence intervals (CIs) for the risk of HUA across different clinical subgroups. Risk factors include overweight, hypertension, dyslipidemia, female sex, diabetes, and osteoporosis. Subgroup analyses stratify risk by sex and metabolic status. *An OR > 1 indicates increased risk, while an OR < 1 indicates decreased risk. The vertical dashed line represents the null effect (OR = 1). *P value less than 0.05, which is statistically significant (i.e., P<0.05). * HUA, hyperuricemia; T2DM, type 2 diabetes mellitus; OR, odds ratio; CI, confidence interval.

Logistic regression analysis (**Figure 5**) identified overweight (OR = 1.76, 95%Cl:1.37-2.26, P<0.001), hypertension (OR = 1.48, 95%Cl:1.17-1.89, P = 0.001), and dyslipidemia (OR = 1.65, 95%CI:1.28-2.12, P<0.001) as independent risk factors. Female sex (OR = 0.20, 95%CI:0.15-0.28, P<0.001), diabetes (OR=0.74, 95%CI:0.56-0.98, P = 0.034), and osteoporosis (OR = 0.62, 95%CI:0.48-0.81, P<0.001) were protective. Subgroup analysis confirmed higher risk in males (32.89% vs. 7.59% events) and metabolic subgroups (overweight/hypertension/dyslipidemia). Age showed no significance (P = 0.921).

## 4 Discussion

It is critical to interpret the statistically significant findings of this exploratory study in the context of their effect sizes. Several associations, while reaching nominal significance, were weak or even trivial in magnitude, underscoring the distinction between statistical significance and clinical meaningfulness.

The prevalence of HUA is 22.86%, This prevalence is consistent with worldwide epidemiological trends, indicating that the local incidence of HUA is higher, which may be related to regional dietary structure, economic development, and lifestyle (1, 4). In the HUA group, the female proportion is significantly lower than males (7.59% vs. 32.89%). Regression analysis showed that female sex was associated with a substantially lower risk of HUA, consistent with previous studies. Ghei et al. attribute the higher SUA levels in males to sex hormone differences (28–30).

Obesity is an important risk factor for HUA. Obesity variables such as BMI and waist circumference (WC) have been shown to be independent predictors of HUA (18, 22, 31). This could be linked to the accumulation of fat cells in obese people, which affects UA production and excretion (32). Animal experiments have shown that adipose tissue can generate and secrete UA via xanthine oxidoreductase (33). Furthermore, obese people frequently have insulin resistance, higher insulin levels, and increased reabsorption of SUA, which reduces UA excretion by the kidney (34–36). In this study, BMI demonstrated a moderate positive correlation with SUA, a weak independent association in linear regression and moderate predictive value in ROC curve analysis.

Hyperlipidemia and HUA are closely related. The results of this study show that HUA group has higher levels of lipid markers such as TG, TC and LDL-C, but lower levels of HDL-C. Correlation analysis revealed a moderate positive correlation for TG and a weak inverse correlation for HDL-C. Multiple linear regression confirmed weak independent associations of SUA with TG. Our findings are consistent with previous evidence (37, 38). A large retrospective cohort study in China indicates that an elevated TG/HDL-C ratio increases the risk of HUA (39). The mechanism linking SUA and TG has not been clarified. However, some viewpoints suggest that SUA can affect lipid metabolism by inducing the production of reactive oxygen species and activating or inhibiting various inflammatory signaling pathways (40). Recent advances in lipidomics research have highlighted the value of integrated omics approaches in deciphering complex lipid metabolism dysregulation and lipid-derived metabolic stress (41), which may provide new insights into the interaction between uric acid and triglyceride metabolism. In addition, some studies explain that HUA and abdominal obesity have a synergistic effect on the probability of developing hypertriglyceridemia and low HDL-C (38). Obesity, a common driver of HUA, MetS, and TG abnormalities, may act synergistically with elevated SUA to increase the risk of hypertriglyceridemia (42). Notably, the absence of independent predictive value for TG in this cohort does not negate its role as a core component of MetS or its established clinical importance in cardiovascular risk assessment—a consensus well supported by extensive evidence.

The study discovered a positive but weak association between DBP and SUA levels, and DBP showed moderate predictive value for HUA. Recent experimental and clinical studies have revealed that increasing SUA levels enhance the risk of hypertension (43, 44). The fundamental mechanisms include the following: Early HUA can cause renal vasoconstriction, which is linked to endothelial dysfunction caused by low levels of nitric oxide (45) and activation of the renin-angiotensin-aldosterone pathway (46). SUA exacerbates endothelial dysfunction and renal microvascular alterations in the early stages of vascular injury by directly promoting smooth muscle and vascular endothelial cell proliferation, as well as having proatherogenic and proinflammatory effects (47–49). Furthermore, UA can stimulate the generation of reactive oxygen species and activate or inhibit a number of inflammatory signaling pathways (40), influencing the development of atherosclerosis. Notably, accumulating evidence indicates that bioactive compounds targeting antioxidant and anti-inflammatory pathways can effectively mitigate cardiometabolic risks by improving endothelial function, supporting overlapping oxidative stress/inflammation pathways between HUA and MetS vascular complications (50). However, while SBP was not significantly associated with SUA levels in correlation analysis, it became negative after multivariate adjustment, which could be due to the influence of other variables included in the adjustment model on the relationship between SBP and SUA, thereby changing the relationship between SBP and SUA.

A meta-analysis of 11 studies previously found that SUA levels were positively linked with the development of T2DM (51). HUA may cause insulin resistance due to endothelial damage and nitric oxide suppression, increasing the risk of diabetes. Meanwhile, increased insulin levels associated with prediabetes can limit uric acid excretion in the kidneys (34, 35, 43). However, in patients with established T2DM, the relationship between chronic glycemia and SUA appears more complex. In our cohort, HbA1c was weakly inversely associated with HUA risk and exhibited no positive discriminative capacity. This finding aligns with large-scale studies demonstrating a bell-shaped (inverted U-shaped) relationship between glycemia and SUA (44, 45), where the association shifts from positive to negative at higher glucose levels. A plausible physiological explanation may involve shared transport pathways for glucose and urate in the renal proximal tubule. Under conditions of significant hyperglycemia, the filtered load of glucose increases markedly. This elevated luminal glucose may interfere with the function of urate transporter 1 (URAT1), which is responsible for apical urate reabsorption (36). Concurrently, systemic and intracellular glucose may influence the activity of glucose transporter 9 (GLUT9), a key facilitator of basolateral urate exit (52). Therefore, high glucose levels may reduce net urate reabsorption by acting on these critical transporters, thereby promoting excretion. It is worth noting that this interaction may also be influenced by renal function status. Clinical studies indicate that the inverse correlation between HbA1c and SUA tends to diminish in patients with decreased eGFR (47). This observation implies that impaired renal function could disrupt the coordinated regulation of glucose and urate transporters, further complicating their association. Future longitudinal studies are warranted to validate the dynamic changes of these relationships during the progression of glucose metabolism disorders and their long-term impact on SUA homeostasis.

SUA levels are regulated primarily by renal excretion. Studies have shown that when renal function deteriorates, SUA level shows an upward trend (48). In this study, the eGFR levels of the participants were within the normal range and no subjects experienced renal failure. As expected, we observed a negative correlation between SUA levels and eGFR.

Furthermore, this study found that the level of SUA was positively correlated with 25(OH)D, implying that a rise in 25(OH)D levels may coincide with an increase in SUA. This outcome is consistent with the findings of the most recent meta-analysis (49). It is important to emphasize that the measured vitamin D metabolite, 25(OH)D, is a storage and transport form, rather than the biologically active hormone 1,25(OH)_2_D. Within this context, the observed positive association may be related to the potential of HUA to modulate renal *CYP27B1* expression, possibly hindering the conversion of 25(OH)D to 1,25(OH)_2_D and thereby elevating 25(OH)D levels (53, 54). Alternatively, elevated SUA linked to renal disorders could alter overall vitamin D metabolism (55). Conversely, urate-lowering therapy has been associated with increased 25(OH)D levels (56), indicating a bidirectional relationship. The interplay among vitamin D status, PTH, and SUA is complex. For instance, concurrent vitamin D deficiency and HUA may exacerbate bone resorption through SUA-mediated pro-oxidative and pro-inflammatory effects (25), although our data suggested an inverse association between osteoporosis and HUA, which may reflect the reverse causality, medication use, or selection bias. Given that our study assessed only the storage form 25(OH)D and did not measure PTH or active 1,25(OH)_2_D, causal mechanistic interpretations are limited. Future research quantifying these active components is required to clarify the underlying pathways.

### 4.1 Limitations

Several limitations should be considered in this study. First, due to its cross-sectional design, causal relationships between SUA and metabolic factors cannot be definitively established. Longitudinal or interventional studies are needed to clarify the direction of these associations. Second, the study participants were recruited from a single center, which may limit the generalizability of the results to other populations or regions. Third, although multiple confounders were adjusted, residual confounding from unmeasured variables (e.g., diet, physical activity, genetic factors) cannot be excluded. Fourth, it is particularly important to note that vitamin D status was assessed only by serum 25(OH)D—a storage and transport form of vitamin D—rather than the biologically active hormone 1,25(OH)_2_D; additionally, PTH was not measured. This lack of data on active vitamin D metabolites and PTH severely limits the mechanistic interpretation of the observed association between SUA and 25(OH)D, as it prevents us from clarifying whether SUA affects the conversion of 25(OH)D to 1,25(OH)_2_D or other potential pathways involved in the vitamin D metabolism axis. Fifth, this is an exploratory analysis with multiple comparisons, which increases the risk of type I error. Although we have reported effect sizes alongside p-values and interpreted them using established thresholds, the findings should be viewed as hypothesis-generating. Finally, medications such as urate-lowering agents or vitamin D supplements were not systematically controlled, which may have interfered with the levels of SUA and 25(OH)D. Future multi-center longitudinal studies with repeated measurements and detailed biochemical and lifestyle data are warranted to validate and extend these findings.

## 5 Conclusion

In summary, this exploratory study provides evidence of significant but predominantly weak-to-moderate associations between SUA and multiple MetS components in Chinese patients with T2DM, with effect sizes interpreted according to established thresholds. BMI, DBP, and 25(OH)D showed moderate predictive value for HUA. Female sex was associated with a substantially lower risk. The negligible correlations with bone metabolism markers and the very weak association with 25(OH)D warrant cautious interpretation. Although TG did not demonstrate independent predictive value for HUA in this cohort, dyslipidemia management remains clinically indicated based on its established role in MetS and cardiovascular risk. These findings underscore the modest interconnections between HUA and metabolic dysregulation.

## Data Availability

The raw data supporting the conclusions of this article will be made available by the authors, without undue reservation.
